# Efficacy and safety of drugs used for ‘assisted dying’

**DOI:** 10.1093/bmb/ldac009

**Published:** 2022-05-04

**Authors:** Ana Worthington, Ilora Finlay, Claud Regnard

**Affiliations:** University of Oxford, Radcliffe Observatory Quarter, Woodstock Rd, Oxford OX1 3PG, UK; Cardiff University, Main Park Place, Cardiff CF14 2TL, UK; St. Oswald’s Hospice, Regent Avenue, Gosforth, Newcastle-upon-Tyne ME3 1EE, UK

**Keywords:** assisted suicide, euthanasia, drug safety, informed consent

## Abstract

**Background:**

‘Assisted dying’ is practiced in some European countries and US states. Legislation suggests that there exists an easily prescribed drug which consistently brings about death quickly and painlessly. Evidence from jurisdictions where ‘assisted dying’ is practiced, however, reveals that hastening patient death is not so simple.

**Sources of data:**

This report is a collation of assisted suicide and euthanasia drug protocols published by the Canadian Association of MAiD Assessors and Providers and the Royal Dutch Medical Association, annual data reports from the USA and Canada and relevant academic publications pertaining to methods of ‘assisted dying’ in the USA, Belgium, Canada and Switzerland.

**Areas of agreement:**

A wide variety of lethal drug combinations are used for people who want their life ended, and the prevalence of complications and failures in intentionally ending life suggest that ‘assisted dying’ applicants are at risk of distressing deaths.

**Areas of controversy:**

The efficacy and safety of ‘assisted dying’ drugs are currently difficult to assess, as clinician reporting is often very low.

**Growing points:**

The findings from this report reveal that little attention has been given to the problem of unmonitored prescribing and administering of lethal drug combinations, whose mode of action is unclear.

**Areas timely for developing research:**

In order to properly assess the efficacy and safety of ‘assisted dying’, a more thorough means of data collection regarding the drugs used must be implemented and research is urgently needed into their mode of action.

‘Assisted dying’ is a legal practice in some countries. Physician-assisted suicide, which licenses clinicians to prescribe lethal drugs for patients to self-ingest, is currently legal in all such legislatures. In addition, euthanasia, in which clinicians inject lethal drugs intravenously to end a patient’s life, is practiced in Belgium, Luxembourg, Canada, New Zealand, Spain, the Netherlands, some Australian states and Colombia.^1^

‘Assisted dying’ legislation often refers to the lethal drugs as ‘medicine’ or ‘medicines’. For example, The Assisted Dying Bill,^2^ a Private Members Bill currently before the UK Parliament, would require patients to confirm: ‘Having considered all this information, I have a clear and settled intention to end my own life and, in order to assist me to do so, I have asked my attending doctor to prescribe medicines for me for that purpose.’ From the use of the term ‘medicines’ it is inferred that there exists an easily prescribed drug that consistently brings about death quickly and painlessly. Evidence from jurisdictions where ‘assisted dying’ is practiced, however, reveals that hastening patient death is not so simple.

No single or combination of drugs is agreed to be most effective for ending a human life. Drugs used for medical purposes are required to undergo a stringent approval process in order to assess efficacy and safety. But the drugs being used for ‘assisted dying’ have not undergone such process; the safety and effectiveness of previous and current combinations of lethal drugs is largely unknown. Canada’s MAiD protocol concedes this.^3^

The pharmacokinetics and pharmacodynamics listed for the medications below are at typical therapeutic dosing, not MAiD dosing. There has been little to no research into their parameters at such high doses as seen with MAiD … There is no peer-reviewed literature to guide best practice in compounding these medications.

There is also evidence that ‘assisted’ deaths may be distressing for patients and their loves ones.^4^^,^^5^

Given that one of the primary aims of introducing ‘assisted dying’ legislation is to provide a ‘safe and comfortable’ death to patients who believe they would otherwise have to endure unbearable suffering at the end of life, it is important to investigate which drugs are being used to bring about patient death as well as the prevalence of complications and failures.^6^ This report examines evidence from jurisdictions where ‘assisted dying’ is practiced in order to ascertain the drugs that are being prescribed and administered, both for physician-assisted suicide (patient ingestion) and for euthanasia (physician administered). Our analysis explored the contents of assisted suicide and euthanasia drug protocols published by the Canadian Association of MAiD Assessors and Providers and the Royal Dutch Medical Association, annual data reports from the USA and Canada and relevant academic publications pertaining to methods of ‘assisted dying’ in the USA, Belgium, Canada and Switzerland. Evidence of complications and failures was also collated alongside popular media coverage which conveys personal experiences of ‘assisted dying’. No new data were generated or analyzed in support of this review.

**Table 1:** Drugs prescribed for assisted suicide, adapted from *Zworth et al. (2020)*^7^

**Description**	**Drug**	**Dose range**
**Sedatives**	Chloral Hydrate	20 g
	Amitriptyline	Not reported
	**Barbiturates**	
	Pentobarbital	9–15 g
	Phenobarbital	20 g
	Secobarbital	9–15 g
	Brallobarbital	Not reported
	Sodium Thiopental	Not reported
	**Benzodiazepines**	
	Diazepam	1 g
	Lorazepam	0.25–2 mg
	Midazolam	10 mg
**Analgesics**	**Opioids**	
	Morphine	15 mg-3 g
	Detropropoxyphene	Not reported
**Cardiotoxic Agents**	Digoxin	50 mg
	Propranolol	2 g
**Antiemetics**	Metoclopramide	10–20 mg
	Ondansetron	8 mg
	Haloperidol	5 mg

**Table 2:** Drugs injected for euthanasia, adapted from *Zworth et al. (2020)*^7^

**Description**	**Drug**	**Dose range**
**Sedatives/Hypnotics**	Propofol	1000–2000 mg
	Vesparax	Not reported
	Chloral Hydrate	35–40 mg
	**Benzodiazepines**	
	Diazepam	10–120 mg
	Lorazepam	1.5–5 mg
	Midazolam	2–120 mg
	**Barbiturates**	
	Pentobarbital	1–15 g
	Thiopental	1–2 g
	Secobarbital	9 g
	Phenobarbital	3000 mg
**Analgesics**	**Opioids**	
	Morphine	16–480 mg
	Fentanyl	25–1500 μg
**Neuromuscular blockers (Paralytic agents)**	MivacuriumAtracuriumAlcuronium	Not reported50–100 mg45 g
	Pancuronium	18–20 mg
	Rocuronium	50–300 mg
	Cisatracurium	30–40 mg
	Vecuronium	10–60 mg
	Curare	Not reported
**Cardiotoxic agents**	Potassium chloride	Not reported
	Bupivacaine	400 mg

We found that a wide variety of lethal drug combinations are used for people who want their life ended, as shown in Table 1 and 2. We also conclude that the prevalence of complications and failures in intentionally ending life suggest that ‘assisted dying’ applicants are at risk of distressing deaths. This holds significant implications for the inclusion of ‘assisted dying’ in clinical practice, and further research is needed into the methods of assisted suicide and euthanasia and the mode of action of drugs used. While individuals may choose to accept that risk, informed choice demands that those risks are understood and clearly explained.

## Drugs used to bring about patient death

### Physician-assisted suicide (oral ingestion)

A scoping review of the drugs used to end life between 1989 and 2020 in the Netherlands, the USA, Belgium, Canada and Switzerland found that a variety of drugs are used including barbiturates, benzodiazepines, sedatives and opioids (Table 1).

The most common lethal drugs used by clinicians to assist suicide were high doses of barbiturates, frequently either pentobarbital or secobarbital. Very high-dose barbiturates have long been a popular method for assisted suicide and are recommended both by the Netherland’s Guidelines for the Practice of Euthanasia and Physician-Assisted Suicide and the Canadian Association of MAiD Assessors and Providers’ oral MAiD medication protocol.^3^^,^^8^

As the availability of these barbiturates has become increasingly scarce and expensive in the USA, alternative drug combinations are now being used. According to Oregon’s 2020 Death with Dignity Act Report, pentobarbital has been unavailable for assisted suicide since 2015, and secobarbital since 2019.^9^ This followed, in part, the December 2011 expansion by the European Commission Regulation on Products used for Capital Punishment and Torture to include products which could be used for the execution of human beings by means of lethal injection.^10^ In the USA, this includes restricting the sale of secobarbital and pentobarbital, drugs frequently used in the execution of prisoners.^11^

In US states where assisted suicide is legal, drug combinations have been used called ‘DDMA’ (diazepam, digoxin, morphine sulfate and amitriptyline) and ‘DDMP’ (diazepam, digoxin, morphine sulfate and propranolol). ‘DDMA’ is the most frequent drug combination used in New Jersey and Oregon, while ‘DDMP’ predominates in Colorado and Hawaii.^9^^,^^12–14^ In the majority of US states where assisted suicide is legal, Washington, California, Washington DC, Maine and Vermont, the drugs used are not recorded.^15–19^

### Euthanasia (physician administered)

In countries where euthanasia is practiced, drug combinations also vary widely and include benzodiazepines, sedatives, neuromuscular blocking agents, opioids and cardiotoxic agents (Table 2).

In the process of euthanasia, practitioners commonly administer a general anesthetic first, frequently a barbiturate or another sedative such as propofol, which seeks to induce unconsciousness. Some also administer an anxiolytic (benzodiazepine) prior to the coma-inducing sedative and, where used, to mitigate propofol-induced pain. After anesthetic, a neuromuscular blocking agent follows. These drugs paralyze all striated muscles, eliminating any movements, both to prevent respiratory effort and to eliminate muscular spasms which could be interpreted as signs of distress by observing relatives. Such regimes can be found in the Guidelines for the Practice of Euthanasia and Assisted Suicide Published by the Royal Dutch Medical Association as well as the Canadian Association of MAiD Assessors and Providers’ intravenous MAiD medication protocol.^8^^,^^20^

## The safety and efficacy of drugs used for ‘assisted dying’

One of the primary purposes of introducing ‘assisted dying’ legislation is to provide a ‘safe and comfortable’ death to patients who believe they will otherwise have to endure unbearable suffering at the end of life.^6^ Fear of future suffering and the desire to control one’s death are among the most prevalent reasons for patients requesting an ‘assisted’ death.^21^^,^^22^ Given these aims, the drugs used should have a high level of efficacy, bringing about death quickly, as well as a high degree of safety, bringing about death without distressing adverse effects.

The efficacy and safety of ‘assisted dying’ drugs are currently difficult to assess. One reason for this is that, in jurisdictions where ‘assisted dying’ is practiced, clinician reporting is often very low. In Belgium, it is estimated that 52% of euthanasia cases are reported to the Federal Euthanasia Control and Evaluation Committee.^23^ In Oregon, patients often ingest the lethal drugs without a healthcare professional present to record complications; a health care professional is reported as present in only one in five such deaths and assisted suicide complications are ‘unknown’ in 71% of cases.^9^ Additionally, data regarding the nature of ‘assisted’ deaths are significantly limited as the reporting mechanisms rely on simple forms filled out only by the prescribing clinician, who may also be reluctant to reveal errors or complications.^24^ Despite limitations in data collection, statistics published in annual reports do reveal that ‘assisted’ deaths are not always accomplished quickly and without complication.

### Physician-assisted suicide (oral ingestion)

Evidence from jurisdictions where assisted suicide is legal reveals that some patients who ingest the prescribed lethal drugs experience distressing complications. The Canadian Association of MAiD Assessors and Providers acknowledges that patients who ingest assisted suicide drugs can experience burning, nausea, vomiting and regurgitation, especially if the patient is experiencing difficulty swallowing large volumes of liquid. Nausea, oesophagitis, gastritis, severe dehydration or pathology of the gastrointestinal tract likely interfere with drug absorption.^3^ This is reflected in the data published by US states such as Oregon, where annual complication rates have been as high as 14.8% and patients are reported to have experienced difficulty swallowing or drug regurgitation, seizures and have even regained consciousness after ingesting the ‘lethal’ drugs.^9^

One reason for such difficulties may be that ingesting sufficiently toxic dosages of the prescribed drugs can prove a significant, and often distressing, challenge. In order to achieve an ‘assisted’ death, patients in the USA have been required to ingest 90 to 100 barbiturate pills by crushing them and mixing them into a sweet solvent. The emetogenic potency and bitterness requires antiemetics prior to ingestion to prevent vomiting. Such an experience of her aunt’s assisted suicide was described by a relative in 2016^4^:

The full ‘cocktail’ included two anti-nausea pills, an anti-seizure pill and 100 capsules of Secobarbital. It all had to be ingested within an hour... My attention turned to the kitchen table, where my husband and sister, wearing latex gloves, frantically scraped the powder from 100 capsules with toothpicks, trying to beat the clock... The mountain of powder we poured into more sugar syrup created a half-cup of sludge so bitter it literally burned my tongue. And my aunt, who could barely swallow water, had to drink all of it in <5 min to ‘ensure success.’... When we sat back down at the kitchen table, white powder everywhere, we all had to wonder, ‘Who the hell wrote this law?’ We had been forced to assist in the most bizarre fashion, jumping through seemingly random legal hoops and meeting arbitrary deadlines while my aunt suffered, and finally emptying capsules, making an elixir so vile I cried when I knew she had to drink it. This was death with dignity?

There is also evidence that the drugs used for assisted suicide do not consistently bring about death quickly. Time to death after ingesting the lethal drugs seems highly unpredictable. Of cases with available data in Oregon since 2001, time from drug ingestion to death has ranged from 1 min (too short for the cause to have been oral drugs) to 108 h. Thirty-three percent of the total deaths with recorded data have taken over an hour, and 7.6% over 6 h. Time to death has become longer since the introduction of experimental drug cocktails ‘DDMA’ and ‘DDMP’. The median time to death after ingestion has doubled since 2015. Fifty-five percent of patients given ‘DDMP2’ (containing 15 g of morphine sulfate) and 45% of those given ‘DDMA’ have experienced a prolonged dying that lasted over 1 h.^9^

In 2017, The Denver Post published an article about a man in Colorado who sought assisted suicide after being diagnosed with cancer. Although his wife thought he would die quickly and peacefully, after ingesting the lethal drugs he experienced distressing complications and took over 9 h to die^5^:

On the day of Kurt’s death, Susan mixed the liquids prescribed as directed and Kurt began drinking the compound. ‘But with every sip,’ Susan says, ‘he’s choking and coughing, choking and coughing.’ It went on for nearly 20 min... Although he never regained consciousness, the gasping, uneven breathing continued. Two hours passed. Then 4 h. ‘At 4:15,’ Susan says, ‘I started to majorly panic.’ As she tried without success to reach a doctor, a couple more disturbing thoughts crossed her mind: She feared that Kurt, despite his unconsciousness, could hear everything—the calls, the desperation in her voice. And she wondered if his choking when he first took the medication meant that he had aspirated enough to delay its effect. Around 7 p.m., she asked hospice to send a nurse. Shortly after the nurse arrived, a doctor called and suggested some additional measures. Soon after, Susan saw her husband sit up slightly and appear to retch three times. She ran to his bedside. Then he slid back into his pillows and stopped breathing.

The unpredictable efficacy of assisted suicide drugs is acknowledged by the Royal Dutch Medical and Pharmaceutical Societies and the Canadian Association of MAiD Assessors and Providers, who both recommend that clinicians obtain consent from patients to convert to euthanasia prior to ingestion of the lethal drugs in case the patient takes too long to die.^8^^,^^25^ In 2018, of the MAiD cases in Canada with available data, 50% were unsuccessful by 60 min and the clinician transitioned to euthanasia to complete the ‘assisted’ death.^25^

### Euthanasia (physician administered)

There are few systematic recordings of complications with euthanasia. Zworth *et al*.’s^7^ scoping review reports that complications with the administration of euthanasia are infrequent but can include difficulty in obtaining or maintaining intravenous access, the person dying too slowly or too quickly, difficulty in pushing a large syringe and pain at the injection site.

However, euthanasia seems to confront a different kind of challenge, as some clinicians question whether death by lethal injection is as peaceful and painless as it may seem. In 2019, a group of clinicians reviewed methods commonly used for euthanasia and contrasted these with an analysis of capital punishment in the USA. Although some of the specific dugs, doses and monitoring techniques employed vary, they found that methods of US capital punishment closely resemble the Dutch injection technique. Their concern lies in the challenge posed by achieving and ensuring unconsciousness during euthanasia, as evidenced from studies of penal death as well as the medical phenomena known as ‘accidental awareness during general anesthesia’.^26^

While caution is needed in extrapolating findings from a generally physically healthy population to those with pathology, based on toxicology reports, up to 88% of executed prisoners studied had postmortem blood concentrations of anesthetic in the blood lower than that required for surgery and 43% had concentrations consistent with awareness.^27^ This means that some prisoners who are subjected to lethal injection do not received a sufficient amount of anesthetic to be fully unaware of the dying process. However, because neuromuscular blocking agents are administered, these prisoners cannot move any muscle even if they remain cognizant and therefore appear to be unconscious.

Sinmyee *et al*.^26^ express concern that monitoring consciousness in patients who are undergoing euthanasia and have received paralytic agents is difficult and, unless properly monitored, ‘there is a risk that vulnerable citizens may be killed by suboptimal, or even cruel, means’. This is of particular concern as opponents of lethal injection argue that a majority of executed prisoners show postmortem signs of flash pulmonary oedema, i.e. their lungs are filled with fluid and weigh several times the weight of normal lungs.^28^ This may be due to toxic drug effects on alveolar basement membrane, allowing rapid fluid ingress. If the person were aware, it has been suggested that the experience of death by lethal injection could be akin to suffocation or drowning.

## Discussion

Clinicians throughout the world are prescribing and administering a wide variety of lethal drug combinations for patients who request an ‘assisted death’. While assisted suicide and euthanasia is often portrayed as a ‘Hollywood’ style peaceful and painless death, evidence from jurisdictions where the practice is legal reveals that this is not always the case. The prevalence of reported and suspected complications suggest there stands a risk of subjecting patients to a less than peaceful death and their loved ones to a traumatic bereavement.

These findings reveal the need for research into the mode of action of drugs used to bring about patient death, as little or no attention has been given to the problems of uncontrolled and unregulated experimentation with drug cocktails which have not been monitored and whose mode of action is unclear. While some countries have medical and pharmaceutical associations that have published recommended ‘assisted dying’ drug protocols, such as the Canadian Association of MAiD Assessors and Providers and the Royal Dutch Medical Association, no government committee funds research on the issued drugs. There is also a need for jurisdictions where assisted suicide and euthanasia is legal to implement more thorough means of data collection regarding the methods of ‘assisted dying’ for inclusion in publicly accessible reports. In many jurisdictions, the drugs and doses prescribed or administered to bring about patient death are not reported.

The prevalence of adverse effects also has implications for clinical practice. The experience of ‘assisted dying’ may not be the ‘safe and comfortable’ process promoted by campaigners, and patients must be properly informed of the realities of hastening death and the risk of distressing complications. In the case of assisted suicide, this includes difficulties ingesting the volume of lethal drugs, adverse reactions to such drugs once ingested, and chances of a prolonged dying which could take several hours. In the case of euthanasia, patients should be informed that cognitive levels can be difficult to monitor and that they will be administered a paralytic agent which will stop them breathing and may inhibit them from alerting anyone if they are in distress. There must also be clear guidelines as to what actions clinicians should take if an ‘assisted death’ goes wrong or fails. Are doctors prepared to be present if the death takes several hours? If the patient regurgitates the drugs, should the clinician clear the airway and put them in the prone position even though such action makes it less likely the patient will die?

As ‘assisted dying’ becomes implemented in Western medicine, the practice must be held to the same standards of any other medical procedure. We could find no reports of systematic monitoring of brain activity until death, blood levels of drugs or postmortem examination of the lungs. As organ harvesting after euthanasia is being practiced in some Benelux countries; we recommend such research be undertaken urgently following assisted suicide and euthanasia.

## Contributors

The conception for this article was a collective effort based on group discussion, with many suggestions circulated by email. AW wrote the first draft, and IF contributed to the methodology and data collation. IF and CR contributed comments and suggestions on subsequent drafts, and AW managed the process of reviews and edits.

## Conflict of interest statement

IF is on the board of Living and Dying Well, a think tank that researches and analyses the evidence surrounding the 'assisted dying' debate. AW declares paid employment with Living and Dying Well for whom she works as a temporary researcher. CR has no interests to declare.
